# Study on 3D High-Resolution Anorectal Manometry Interrater Agreement in the Evaluation of Dyssynergic Defecation Disorders

**DOI:** 10.3390/diagnostics13162657

**Published:** 2023-08-11

**Authors:** Justin Y. van Oostendorp, Pieter van Hagen, Grietje J. H. van der Mijnsbrugge, Ingrid J. M. Han-Geurts

**Affiliations:** 1 Proctos Kliniek, Prof. Bronkhorstlaan 10, 3723 MB Bilthoven, The Netherlands; p.vanhagen@proctoskliniek.nl (P.v.H.); gjh.vandermijnsbrugge@proctoskliniek.nl (G.J.H.v.d.M.); i.han@proctoskliniek.nl (I.J.M.H.-G.); 2 Department of Surgery, Amsterdam University Medical Centers, Location AMC, Meibergdreef 9, 1105 AZ Amsterdam, The Netherlands

**Keywords:** three-dimensional high-resolution anorectal manometry, 3D-HRAM, defecation disorders, chronic constipation, fecal incontinence, dyssynergia, interrater agreement

## Abstract

Anorectal manometry measurements exhibit significant interrater variability. Newer techniques like 3D high-resolution anorectal manometry (3D-HRAM) have the potential to enhance diagnostic accuracy and our understanding of defecation disorders. However, the extent of interrater variability in 3D-HRAM is still unknown. Between January 2020 to April 2022, patients referred for pelvic floor physical therapy (PFPT) due to functional defecation complaints underwent 3D-HRAM testing. In a retrospective analysis, three expert raters independently evaluated the 3D-HRAM results in a blinded matter to assess interrater agreement. The evaluation also determined the level of agreement concerning dyssynergic patterns during simulated defecation. The 3D-HRAM results of 50 patients (37 females) were included. Twenty-nine patients had complaints of fecal incontinence, eleven patients had chronic constipation, and ten patients had several other complaints. There was a substantial agreement (kappa 0.612) between the raters concerning the 3D images on dyssynergic patterns during simulated defecation. Our study emphasizes the need for standardized guidelines in evaluating 3D-HRAM test results to reduce subjectivity and further improve agreement among raters. Implementing these guidelines could improve diagnostic consistency and enhance personalized treatment strategies, increasing the reliability and usefulness of 3D-HRAM testing in clinical practice.

## 1. Introduction

Chronic constipation and fecal incontinence are functional defecation disorders that affect people of all ages and are associated with a reduced quality of life [1,2]. Together, they have a pooled prevalence of 16% in the general population, with higher rates observed in women and the elderly [3,4,5]. These disorders also represent a significant economic burden, including increased healthcare utilization and costs [6].

Functional defecation disorders present several overlapping symptoms, thereby complicating accurate diagnosis and potentially resulting in a wide spectrum of treatment modalities accompanied by significant challenges. It can be time-consuming and frustrating for both the patient and the treating specialist; therefore, it is essential to correctly identify the underlying cause.

Conservative treatment that includes lifestyle changes, exercise, an increase in the intake of dietary fiber, laxatives, and pelvic floor physical therapy is usually sufficient to reduce or eliminate symptoms in the majority of patients [7]. However, when conservative treatment is unsuccessful, additional diagnostic tests such as surface electromyography (s-EMG), anorectal manometry (ARM), imaging studies, or colonoscopy may be necessary to determine the underlying cause of the symptoms. 

Functional defecation disorders are defined by the Rome IV criteria as disordered evacuation resulting from an inadequate recto-anal pressure gradient brought on by paradoxical contraction or insufficient relaxation of the pelvic floor muscles and/or insufficient rectal propulsive forces during defecation [8,9]. Dyssynergic defecation can only be officially diagnosed when the following three conditions are met: (1) symptoms of functional constipation (Rome IV), (2) a pattern of dyssynergic defecation revealed via ARM or s-EMG, and (3) at least one additional quantifiable abnormal defecation measure, such as an abnormal balloon expulsion test (BET), prolonged delay in colonic transit, or incomplete evacuation during defecography [10]. 

Diagnostic tests such as three-dimensional high-definition anorectal manometry (3D-HRAM) or defecography can provide valuable insight into the underlying cause of anorectal dysfunction and guide the development of a tailored treatment plan. Which anorectal function test is the most accurate is still under debate. In our previous study, we demonstrated that for detecting dyssynergic defecation, there is no correlation between 3D-HRAM interpretation and other anorectal function tests such as DRE (52% agreement), transperineal ultrasound (25% agreement), s-EMG (52% agreement), or a balloon expulsion test (46% agreement) [11]. Other studies have reported similar results [12,13]. 

ARM is a diagnostic tool used to evaluate the function of the rectum and anal sphincter muscles in an objective manner. It involves inserting a catheter into the rectum and measuring the pressure and sensation in the anal canal at rest, during squeezing, and simulated evacuation. Traditional anorectal manometry uses a single sensor to measure pressure, while 3D-HRAM uses an array of 256 sensors, providing a more detailed evaluation of the anorectal anatomy and function thanks to its 3D pressure profile map. 3D-HRAM is a valuable diagnostic tool for evaluating pelvic floor dysfunction in patients experiencing obstructive defecation or fecal incontinence [14]. It can identify specific areas of muscle dysfunction and structural or sensory abnormalities, and theoretically detect specific patterns of dysfunction, such as dyssynergic defecation, which is a major cause of functional defecation disorders [15].

Despite its usefulness in evaluating anorectal function, measurements obtained during ARM can be subject to substantial external influence [16,17]. A recent retrospective study demonstrated significant inter-operator variance in HRAM results, despite using similar patient instructions [18]. This variance led to different diagnoses and treatment strategies in a significant number of patients. Moreover, conventional anorectal manometry cannot differentiate between healthy individuals and those with obstructed defecation complaints, particularly in relation to dyssynergic defecation [19]. 

3D-HRAM has been shown to enhance diagnostic gain by visualizing puborectal muscle function and identifying focal sphincter defects [20]. Despite the potential benefits of 3D-HRAM, the accuracy and reliability of its results depend on the skill and expertise of the interpreting rater. A recent study demonstrated that different blinded raters showed only fair interrater variation in identifying dyssynergic patterns, with a 63% agreement in presence detection (Cohen’s kappa 0.340) [20]. Dyssynergic patterns are also observed in asymptomatic, healthy subjects, which contributes to the complexity [21]. Therefore, it seems crucial to have experienced raters interpreting 3D-HRAM results to ensure consistency and accuracy and avoid misdiagnosis. 

The question of whether we can rely on 3D-HRAM results to interpret anorectal function and guide treatment strategies is complex, as both operator variability and interrater variability can affect the reliability and diagnostic accuracy of the test [18,22,23]. The present study aimed to investigate the interrater agreement of 3D-HRAM results between experienced clinicians working in a specialized Dutch tertiary referral clinic for proctology. This study aims to better understand the extent to which different expert raters interpret 3D-HRAM results.

## 2. Materials and Methods

### 2.1. Study Design and Population

This retrospective study was conducted at a Dutch tertiary referral clinic specialized in proctology, where a multidisciplinary team consisting of surgeons, gastroenterologists, pelvic floor physiotherapists, and specialized nurses work closely together in the treatment of patients with defecation disorders. Recently, we published the findings of a study by Dekker et al., which involved fifty patients who consented to participate [11]. These patients presented with complaints of fecal incontinence or chronic constipation between January 2020 and April 2022 and were referred by the surgeon to the pelvic floor physiotherapist for diagnostic evaluation and treatment. Patients were recruited consecutively, and the eligibility criteria have previously been described elsewhere [11]. For this study, patients underwent a number of anorectal function tests, aiming to establish an association between them. The 3D-HRAM test results were selected for this subsequent analysis.

Patient data including demographics, complaints, previous perianal surgical procedures, obstetric history, and history of previous pelvic floor physical therapy were collected. To evaluate the interrater agreement of 3D-HRAM results, three experienced raters (two surgeons and one gastroenterologist) independently evaluated the results of fifty patients. To ensure unbiased evaluation, the raters were blinded to the initial reports and to the evaluations of the other raters. To evaluate intra-rater agreement, two raters evaluated several cases at two different time points: once as part of the diagnostic process and once for the present study, with a minimum interval of 6 months between these two evaluations. The raters were also blinded to their original reports to prevent any bias in the evaluation.

### 2.2. Three-Dimensional High-Resolution Anorectal Manometry (3D-HRAM)

For 3D-HRAM, the equipment consists of the following components: a probe, a pressure recording device, a monitor, and a computer used for data storage. The 3D-HRAM probe is equipped with 256 pressure sensors on 16 lines, with each line containing 16 circumferential sensors. It is covered by a disposable sheath, measuring 10.75 mm in diameter and 64 mm in length, which has an inflatable balloon (capacity 400 cc) at the most distal end.

Two experienced continence nurse specialists operated the 3D-HRAM in a protocolized matter, following the International Anorectal Physiology Working Group (IAPWG) protocol [24]. The instructions and verbal feedback given during the test follow the recommendations of the IAPWG and were the same for all subjects [17,24]. Patients underwent the examination in a left lateral position with flexed hips and knees and were instructed to use an enema the night before and in the morning before the examination. Pressures were recorded at rest, during squeezing, and while straining, in accordance with the standardized IAPWG protocol [24]. The obtained manometry data were subsequently analyzed using ManoView software (Given Imaging, Duluth, GA, USA) using both 2D and 3D images.

### 2.3. Study Parameters

**Figure 1:**
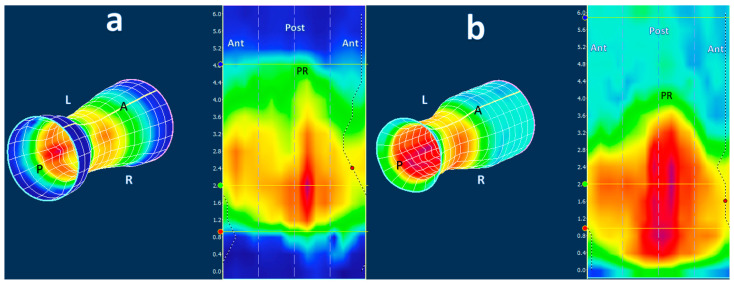
Example of the pressure profile in 2D and 3D using ManoView software of a 70-year-old female with chronic constipation: (**a**) elevated anal resting pressure and (**b**) a dyssynergic pattern during simulated defecation characterized by paradoxical contraction of the pelvic floor muscles. Legend: The puborectal muscle (PR) is seen in the middle of the 2D profile. ATN = anterior, POST = posterior, L = left, R = right. The white line in the cylinder is ‘cut’ and unrolled to obtain the 2D image, so that the anterior is on both sides (ANT) of the 2D image.

**Figure 2:**
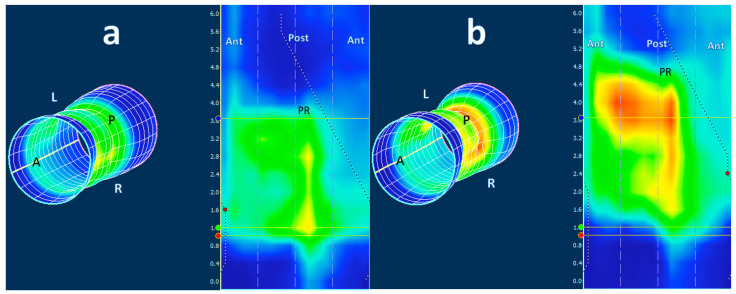
Example of the pressure profile in 2D and 3D using ManoView software of a 45-year-old male with fecal incontinence. (**a**) Low anal resting pressure and (**b**) signs of reduced contractility, particularly on the anterior side (shown in blue), characterized by a diminished ability of the IAS muscles to generate a coordinated contraction. Legend: The puborectal muscle (PR) is seen in the middle of the 2D profile. ATN = anterior, POST = posterior, L = left, R = right. The white line in the cylinder is ‘cut’ and unrolled to obtain the 2D image, so that the anterior is on both sides (ANT) of the 2D image.

The software calculated the mean resting pressure (MRP) and maximal squeeze pressure (MSP) in each patient, which are standard parameters in HRAM manometry. Meanwhile, the 3D-HRAM-derived images were collected and compiled into a single database with all patient identifiers removed. Visual representations of the changes in anal and rectal pressure during the protocol maneuvers are presented through color patterns and 2D and 3D images (Figure 1 and Figure 2). The pressure zones are color-coded, with purple indicating the highest pressure, followed by red, yellow, green, and finally blue, representing areas of low pressure at specific time points.

The color contour plots of the 2D- and 3D-HRAM images were evaluated by three independent raters (IHG, RFB, GVM) and categorized as low, normal, or high pressure, as indicated in Table 1. To enhance accuracy and minimize measurement variability obtained through the software, three measurements were captured and then averaged. The mean resting pressure provides valuable insights into anal sphincter hypo- or hypertension, while the maximal squeezing pressure indicates sphincter contractility. Additionally, we measured the functional length of the anal canal, as a shorter anal canal could be associated with anal hypotonia.

During simulated defecation (straining), we measured several parameters including mean anal resting pressure, percentage of anal relaxation, intra-rectal pressure, and recto-anal pressure gradient (RAPG). Additionally, we calculated the percentage of positive RAPG, which could suggest adequate rectal propulsive forces during simulated defecation. Furthermore, the three raters independently evaluated the 2D and 3D images for the presence of a dyssynergic pattern of defecation during straining, defined as paradoxical anal contraction (an increase in anal sphincter pressure), inadequate relaxation of the resting anal sphincter pressure, or inadequate effort of the abdominorectal muscles [9]. In normal defecation, the subject generates adequate intra-abdominal pressure and push force, along with adequate relaxation of the anal sphincter pressure. The intra-rater agreement was based on the evaluation of two blinded raters at different time points.

Finally, the presence of slow waves and ultraslow waves (USWs) was evaluated visually by two of the three independent raters. These two raters conducted a visual evaluation and classification of maximum tolerated volume (MTV) sizes, along with the measured values of the first constant sensation volume (FCSV) and desire to defecate volume (DDV). This analysis aimed to determine whether these factors contributed to any sensory disorders.

### 2.4. Statistical Analysis

Statistical analyses were performed using SPSS software (IBM, Armonk, NY, USA, SPSS Statistics 28). Continuous data are reported as either median or mean, depending on the distribution, accompanied by the range or standard deviation. For categorical outcomes from the 3D-HRAM evaluation, descriptive statistics and crosstabs were used for comparison. The interrater agreement among the different raters for various 3D parameters, including mean resting pressure, maximum squeeze pressure, dyssynergia, presence of (ultra)slow waves, rectal volume, and sensibility, was evaluated using Cohen’s Weighted Kappa test [25]. The agreement was classified according to established criteria: poor (0–0.20), fair (0.21–0.40), moderate (0.41–0.60), substantial (0.61–0.80), and almost perfect agreement (0.81–1.00), as reported in previous studies [19,20]. Statistical significance was considered as *p* < 0.05. 

## 3. Results

### 3.1. Patient Demographics and Clinical Characteristics

A total of 50 patients (13 males, 37 females) with a median age of 51 years were included in this analysis. The indication for 3D-HRAM differed for these 50 patients: 29 patients had complaints of fecal incontinence, 11 patients had chronic constipation, and 10 patients had various other anorectal complaints (Table 2). Thirty of the thirty-seven females (81%) had one or more vaginal deliveries. In total, 19 patients (38%) underwent rectal, urologic, or gynecologic surgery in the past. Thirty-one patients (62%) were previously treated via pelvic floor physical therapy (PFPT).

### 3.2. Descriptive Statistics of the 3D-HRAM Software Measurements

The descriptive statistics of the 3D-HRAM software-derived data of the pressure variables in the study group are detailed in Table 3. The median length of the anal canal was 31 mm (IQR 12.5). In twenty-six patients (52%), the anal canal length was below average (normal value 33–45 mm). The median resting pressure was 67 mmHg (SD 30) and median maximum squeeze pressure was 139 mmHg (SD 84). During the push maneuver, the median pressure in the rectum did not exceed the pressure of the anal canal. The RAPG was found to be positive (>0 mmHg) in only 10 patients (20%), indicating a positive propulsive force during simulated defecation. The median percentage of anal relaxation was 35% (SD 17). The MTV was 150cc (SD 71).

### 3.3. Interrater Agreement

The evaluation of anal resting pressures revealed a significant level of agreement among the three raters, with a mean kappa of 0.512 (*p* < 0.001). However, for maximum squeeze pressures, the agreement was only deemed fair, with a mean kappa of 0.329 (*p* < 0.001). The evaluation of a dyssynergic pattern during three straining maneuvers (simulated defecation) resulted in a substantial level of agreement, with a mean kappa of 0.612 (*p* < 0.001). In terms of (ultra)slow waves and MTV size and sensibility, the agreement was found to be poor, almost perfect, and substantial, respectively (Table 4).

### 3.4. Intra-Rater Agreement

Two independent raters evaluated 39 individual 3D-HRAM results at two different time points, with a minimum interval of 6 months between the two time points, blinded to their original reports. Regarding the presence of a dyssynergic pattern during simulated defecation, there was an intra-rater agreement of 87.2%.

## 4. Discussion

Anorectal manometry is a tool for diagnosing functional defecation disorders. The introduction of 3D-HRAM has significantly enhanced diagnostic accuracy through incorporating 3D imaging capabilities. It enables an overview of a comprehensive pressure map of the entire anal canal, providing valuable insights into the underlying pathophysiology.

In this study, we demonstrated that between three expert raters who are proficient in interpreting 3D-HRAM results, there was a moderate level of agreement when evaluating patients with defecation disorders. Specifically, when evaluating anal resting pressures and maximum squeezing pressures (categorizing them as low, normal, or high), we observed moderate or fair agreement, respectively. These findings indicate that there is a significant amount of variation in the interpretation of 3D-HRAM results.

The variability in agreement regarding conventional HRAM results might be attributed to the reliance on software-derived values, which often leads to the interpretation of mean pressures at rest and during squeezing and straining. However, when examining the 3D images, it becomes evident that there are regions of high pressure in a specific area of the anal sphincter, indicating hypertonia, while simultaneously showing a defect in another area, resulting in low pressure at that specific point. This information is crucial because solely looking at mean pressure values can thus lead to the incorrect conclusion that there is no pathology, potentially affecting the treatment strategies and conclusions drawn for patients.

The evaluation of a dyssynergic pattern during three subsequent straining maneuvers demonstrated substantial agreement among the three raters. However, challenges were observed in visually evaluating dyssynergia in patients with low anal resting tones, probably due to less pronounced patterns. Unlike previous studies, the raters categorized the presence of a dyssynergic pattern as either yes or no, without using different categories or phenotypes [19,20]. Previous studies have identified various patterns of dyssynergic defecation, contributing to functional defecation disorders [19,24,26,27]. It was found that an insufficient increase in rectal pressure during straining (<45 mmHg) could be indicative of dyssynergic defecation. Also noteworthy, high anal pressures during straining were not necessarily linked to pathology, as even healthy individuals showed elevated pressures [19]. However, the presence of anal sphincter dyssynergy, along with a high resting anal pressure (>92 mmHg), may potentially indicate pathology [19]. So, anal sphincter dyssynergia alone is not considered a pathological finding unless accompanied by elevated resting tone. Some systems use the cutoff value of >92 mmHg to define high resting pressure, which appears to be a valid threshold [19].

In the literature, ultraslow waves are defined as repeated pressure oscillations occurring at a rate of 1–2 per minute with an amplitude >25 mmHg, while slow waves occur at a rate of 10–20 per minute and are also widely present in healthy people [7,14]. The role and implications of USWs are still unclear. The presence of USWs on 3D-HRAM images might suggest internal anal sphincter (IAS) hypertonicity [7]. While the clinical significance of this finding remains undetermined, it might offer valuable insight into the underlying pathophysiology and aid in determining appropriate treatment strategies (e.g., Botox injection for patients experiencing obstructed defecation due to IAS hypertonicity). However, our study revealed poor agreement among raters regarding the presence of (ultra)slow waves on 3D images. Therefore, it is necessary to establish standardized evaluation criteria. 

Our study suggests that a more homogeneous patient population leads to higher agreement levels among raters. In a sub-analysis of 3D-HRAM results from patients suffering from chronic constipation (*n* = 11), we observed almost perfect agreement among raters regarding dyssynergic defecation patterns (kappa = 0.855). Therefore, it is crucial to create a clear overview of different patient groups and their symptoms, incorporating 3D-HRAM results to gain a better understanding of the underlying pathophysiology. It is important to note that in this specific test cohort, the raters were intentionally blinded to patients’ history, complaints, and digital rectal examination findings. The aim was to solely evaluate interrater agreement based on the 3D-HRAM results. However, in real-world diagnostic scenarios, this additional information would be considered and integrated with the 3D-HRAM results, providing a comprehensive and more accurate diagnostic evaluation.

Several studies have examined the normal values of 3D-HRAM in healthy individuals and proposed cutoff values based on the 95% confidence interval [21,28,29,30]. According to these cutoffs, a mean resting pressure (MRP) below 50 mmHg is considered low pressure, while a mean resting pressure above 100 mmHg and a maximum squeeze pressure above 200 mmHg are classified as high pressure. These studies highlight the impact of age, parity, and gender on the range of values observed [28,29,30,31,32,33]. Also, the IAPWG refrained from providing specific normal values due to variations in equipment, protocols, and practices [24].

Standardizing the evaluation of 3D-HRAM test results is crucial to establish universally agreed-upon normal values and patterns. Unlike other manometry techniques, which lack standardization and reliability, high-resolution anorectal manometry offers more precise measurements due to advancements in sensor technology and software metrics, as well as the visual evaluation provided by 3D images. However, it is important to regard 3D-HRAM as a complementary tool, used in conjunction with other diagnostic tests such as balloon expulsion or defecography, to accurately evaluate the underlying causes of symptoms and guide treatment strategies.

As we transition towards a more personalized approach to treatment, particularly in patients with functional defecation disorders where treatment choices can be challenging, it becomes increasingly valuable to standardize evaluation and classify different functional phenotypes. The IAPWG has introduced the London classification for disorders of anorectal function, with Type 3 disorders focusing on recto-anal coordination issues and encompassing various dyssynergic patterns [24]. These patterns involve evaluating propulsive force adequacy and the relaxation or contraction of the anal sphincter during defecation. This classification provides valuable insights into the underlying causes of the problem, assisting in the selection of appropriate treatment options. Consequently, 3D-HRAM can serve as a cornerstone in the management of these patients.

This study is the first to evaluate the agreement among experienced raters in a blinded and independent manner regarding 3D-HRAM test results for patients with functional defecation disorders. However, it is important to acknowledge the study’s limitations. We report on a retrospective sub-analysis of a single-center modestly sized cohort (*n* = 50), with each rater performing evaluations in their own individual manner without a standardized approach. Additionally, intra-rater data was available for only a subset of patients (*n* = 39). Since there was no established gold standard, reference test, or consensus agreement, it was challenging to compare the outcomes and accuracy of the three evaluations. 

The agreement regarding dyssynergic patterns in 3D-HRAM is significant. Looking ahead, there are promising prospects for utilizing 3D-HRAM in other medical indications. Notably, it has shown potential in the field of fistula diagnostics [34]. In the future, incorporating 3D-HRAM results might help reduce the risk of inducing incontinence during fistula surgeries, particularly fistulotomy. Ongoing research at our clinic is actively investigating this area to delve deeper into the subject and expand our understanding.

## 5. Conclusions

This study revealed that the current approach for the evaluation of 3D-HRAM test results, including the interpretation of 3D images, is subjective and leads to significant disagreement among experienced raters. Therefore, it is crucial to establish standardized guidelines for evaluating 3D-HRAM test results, preferably incorporating patterns categorized by specific functional phenotypes. This approach can help clinicians minimize subjectivity and enhance the uniformity of diagnoses. This standardization has the potential to enhance treatment strategies, ensuring they are more personalized to meet the individual needs of patients. Ultimately, implementing standardized guidelines may increase the reliability and usefulness of 3D-HRAM testing in clinical practice.

## Figures and Tables

**Table 1:** Summary of 3D-HRAM categories and their potential outcomes.

Mean Resting Pressure	Maximum Squeezing Pressure	Push Maneuver	Slow Waves	Ultra Slow Waves	MTV ^1^	Sensibility
1. Low	1. Low	1. Relaxation	1. Yes	1. Yes	1. Small	1. Normal
2. Normal	2. Normal	2. Paradoxal	2. No	2. No	2. Normal	2. Hyposensitive
3. High	3. High				3. Big	3. High

^1^ MTV = maximum tolerated volume.

**Table 2:** Characteristics of the study population.

Descriptives	Number of Patients
Gender	
Male, *n* (%)	13 (26)
Female, *n* (%)	37 (74)
Median age, years (IQR)	51.5 (21.25)
Indication, *n* (%)	
Fecal incontinence	29 (58)
Chronic constipation	11 (22)
Chronic anal fissure	4 (8)
Hemorrhoidal disease	2 (4)
Other	4 (8)
Vaginal parity, *n* (%)	
0	7 (19)
1	7 (19)
2	14 (38)
≥3	9 (24)
Rectal surgery in the past, *n* (%)	9 (18)
Radiotherapy in the past, *n* (%)	1 (2)
Urologic or gynecologic surgery in the past, *n* (%)	10 (20)
Neurological or connective tissue disease, *n* (%)	3 (6)
Previous pelvic floor physical therapy, *n* (%)	31 (62)

**Table 3:** Software-derived data for defecatory pressure variables. mmHg = millimeters of mercury; IQR = interquartile range; n/a = not applicable.

Measurements 3D-HRAM Software	Medians (IQR)	Normal Values
Median length anal canal, mm	33 (12.5)	33–45
Median resting pressure, mmHg	67 (46.4)	Female: 60–85 Male: 60–85
Median maximal squeeze pressure, mmHg	139 (132)	Female: 85–135 Male: 115–200
Median anal rest-pressure during push, mmHg	+49 (43.8)	n/a
Median percentage anal relaxation, %	34.5 (22.2)	50–100%
Median intra-rectal pressure during push, mmHg	+22.5 (30.5)	n/a
Median Recto-Anal Pressure Gradient (RAPG), mmHg	−21.3 (59.2)	n/a
Recto-anal pressure gradient (RAPG), *n* (%)		n/a
Negative	40 (80)	
Positive	10 (20)	
Median Maximal Tolerable Volume (MTV), cc	150 (105)	145–230 cc
Median First sensation, cc	50 (45)	20–45 cc

**Table 4:** Interrater agreement after visual evaluation of 3D-HRAM results by 3 independent raters.

Category	Raters	Kappa Agreement	*p*-Value
Resting pressure	1 vs. 2 1 vs. 3 2 vs. 3 Mean	0.649 0.473 0.413 0.512	<0.001 <0.001 <0.001 <0.001
Squeezing pressure	1 vs. 2 1 vs. 3 2 vs. 3 Mean	0.235 0.464 0.288 0.329	0.005 <0.001 0.004 <0.001
Dyssynergia	1 vs. 2 1 vs. 3 2 vs. 3 Mean	0.634 0.655 0.547 0.612	<0.001 <0.001 <0.001 <0.001
Slow waves	1 vs. 3	−0.008	0.942
Ultra slow waves	1 vs. 3	0.122	0.184
MTV categorical	1 vs. 3	0.843	<0.001
Sensibility categorical	1 vs. 3	0.641	<0.001

## Data Availability

Our data are available on reasonable request.
